# Population Evolution, Genetic Diversity and Structure of the Medicinal Legume, *Glycyrrhiza uralensis* and the Effects of Geographical Distribution on Leaves Nutrient Elements and Photosynthesis

**DOI:** 10.3389/fpls.2021.708709

**Published:** 2022-01-07

**Authors:** Hanli Dang, Tao Zhang, Yuanyuan Li, Guifang Li, Li Zhuang, Xiaozhen Pu

**Affiliations:** ^1^College of Life Sciences, Shihezi University, Shihezi, China; ^2^Key Laboratory of Oasis Eco-Agriculture, College of Agriculture, Shihezi University, Shihezi, China

**Keywords:** whole genome re-sequencing, population evolution, genetic diversity, adaptive evolution, *Glycyrrhiza uralensis*

## Abstract

*Glycyrrhiza uralensis* is a valuable medicinal legume, which occurs widely in arid and semi-arid regions. *G. uralensi*s demand has risen steeply due to its high medical and commercial value. Interpret genome-wide information can stimulate the *G. uralensis* development as far as its increased bioactive compound levels, and plant yield are concerned. In this study, leaf nutrient concentration and photosynthetic chlorophyll index of *G. uralensis* were evaluated to determine the *G. uralensis* growth physiology in three habitats. We observed that *G. uralensis* nutrient levels and photosynthesis differed significantly in three regions (*p* < 0.05). Whole-genome re-sequencing of the sixty *G. uralensis* populations samples from different habitats was performed using an Illumina HiSeq sequencing platform to elucidate the distribution patterns, population evolution, and genetic diversity of *G. uralensis*. 150.06 Gb high-quality clean data was obtained after strict filtering. The 895237686 reads were mapped against the reference genome, with an average 89.7% mapping rate and 87.02% average sample coverage rate. A total of 6985987 SNPs were identified, and 117970 high-quality SNPs were obtained after filtering, which were subjected to subsequent analysis. Principal component analysis (PCA) based on interindividual SNPs and phylogenetic analysis based on interindividual SNPs showed that the *G. uralensis* samples could be categorized into central, southern, and eastern populations, which reflected strong genetic differentiation due to long periods of geographic isolation. In this study, a total of 131 candidate regions were screened, and 145 candidate genes (such as Glyur001802s00036258, Glyur003702s00044485, Glyur001802s00036257, Glyur007364s00047495, Glyur000028s00003476, and Glyur000398s00034457) were identified by selective clearance analysis based on Fst and θπ values. Gene Ontology (GO) and Kyoto Encyclopedia of Genes and Genomes (KEGG) pathway analysis showed significant enrichment of 110 GO terms including carbohydrate metabolic process, carbohydrate biosynthetic process, carbohydrate derivative biosynthetic process, and glucose catabolic process (*p* < 0.05). Alpha-linolenic acid metabolism, biosynthesis of unsaturated fatty acids, and fatty acid degradation pathways were significantly enriched (*p* < 0.05). This study provides information on the genetic diversity, genetic structure, and population adaptability of the medicinal legumes, *G. uralensis*. The data obtained in this study provide valuable information for plant development and future optimization of breeding programs for functional genes.

## Introduction

The liquorice (*Glycyrrhiza uralensis*) is a widely consumed medicinal plant. It is a type of leguminous perennial herb that is widely distributed in many arid and semi-arid regions of the world, including China (Liu et al., 2020). The *G. uralensis* species is a drought-tolerant and deep-rooted plant that plays a crucial role in the ecosystems of desert and semi-desert areas of Northwest China by participating in the windbreak, sand fixation, and soil nutrient conservation (Liu et al., 2007). *G. uralensis* dried roots contain a myriad of bioactive compounds, including triterpenoid saponins, polysaccharides, and flavonoids (Niu et al., 2009; Wang et al., 2015). Thus, they are widely used in clinical medicine due to their anti-inflammatory, immunomodulatory, and antiviral activities (Liu et al., 2010; Hosseinzadeh and Nassiri-Asl, 2015). In addition, as *G. uralensis* is sweeter than sucrose, it is also used as a sweetener and food additive in the food industry (Seki et al., 2011). Although the content of bioactive components extracted from *G. uralensis* leaves is lower than that from roots, it is rich in fat, proteins, and trace elements, which enables it to meet the nutritional requirements of ruminants (Liu et al., 2013). Therefore, *G. uralensis* leaves can be used as feed or feed additives as they do not have toxic side effects and drug resistance. Besides, their high nutritional composition improves animal health, promotes growth, enhances animal immunity, and improves animal production performance, making them good forage grass resources in animal husbandry with considerable economic benefits (Su et al., 2010).

Genetic diversity is an important index for discerning the composition of alleles and genotypes of populations, also a major tool to explore the genetic relationships and evolutionary trends among populations (Muccillo et al., 2019). Geographical isolation and long-term interaction with different natural habitats have resulted in remarkable differences in morphological characteristics, physiological responses (Farhadi and Omidbeygi, 2014), active component content (Yang et al., 2018), and genetic material of natural *G. uralensis* among populations (Techen et al., 2015). Multiple studies (Wen-Bin et al., 2019) have shown that the morphological changes in natural *G. uralensis* among populations are mainly focus on plant height, flowers, fruit pods, and leaves, among which the leaf morphology is greatly affected by the environment, which is closely related to the response of leaf epidermis (stomatal size, stomatal density, and stomatal index) to geographical differences in habitats, including light factor, temperature factor, and water factor (Lu et al., 2011). In addition, the main environmental limiting factors, including water, light and salt stress, resulted in significant differences in physiological and biochemical indices (biomass, photosynthetic pigment content, peroxidase, superoxide dismutase) and secondary metabolites (glycyrrhizic acid, isoliquiritigenin, and liquiritin) contents among *G. uralensis* natural populations (Liu, 2006; Li et al., 2011; Hosseini et al., 2019). There is rich genetic variation in *G. uralensis* natural populations, which has been widely demonstrated in DNA molecular studies. For example, Ge et al. (2009) based on amplified fragment length polymorphism (AFLP) markers reported extensive genetic variation within the wild *G. uralensis* population in China, and it was significantly related to the material’s geographical distribution. Liu et al. (2019) based on transcriptome simple sequence repeat (SSR) markers reported higher genetic variation among *G. uralensis* natural populations than between *G. uralensis* species. These studies provided a theoretical basis for the improved breeding and conservation of wild *G. uralensis* plants.

Previously, the genetic structure and diversity of *G. uralensis* populations were determined using the microsatellite markers (Yang et al., 2016) and single nucleotide polymorphism (SNP) (Liu et al., 2019). However, insufficient sample size or uneven distribution of markers has restricted the understanding of genetic diversity and genetic regions of the whole genome of medicinal *G. uralensis* species. The currently available whole-genome sequence of ‘*G. uralensis* (Mochida et al., 2017) has facilitated the whole genome re-sequencing of *G. uralensis* species from different geographical areas to elucidate *G. uralensis* genetic differentiation and adaptive evolution of whole genome, which has not been reported to date.

Whole-genome re-sequencing (WGR) of plant genomes can detect an array of DNA, such as single nucleotide polymorphisms (SNPs), insertions and deletions, copy number variation (CNV), and presence/absence variation (PAV) (Huang et al., 2012; Jiao et al., 2012; Xu and Bai, 2015), providing a basis for an in-depth understanding of genomic evolution. Sequence alignment can detect genetic and intergenic variations, including some non-synonymous mutations in coding or regulatory regions, which are crucial for studies related to gene function and genetic differentiation (Boycheva et al., 2014; Nützmann and Osbourn, 2014).

With recent advances in sequencing technology, whole genome sequencing and large-scale re-sequencing were applied to investigate multiple plant species. For instance, mutation sites in cotton (Shen et al., 2017), genome association analysis of rice (Wang et al., 2016), domestication, and agronomic trait detection of Pigeon pea (Varshney et al., 2017) were investigated using genomic sequencing. Intensive research activities have accurately assessed the extensive patterns of genetic variation and population differentiation across multiple related species (Ellegren et al., 2012; Feulner et al., 2015), and the outcomes of these studies have broadened researchers’ understanding of the application and understanding of genetic variation and adaptation in plants. Although the medicinal and commercial demand for *G. uralensis* species has increased rapidly, *G. uralensis* plantation number and genome-wide information have hardly progressed. This, in turn, has adversely affected the research efforts related to increasing the concentration of bioactive compounds and productivity of *G. uralensis* plants through molecular breeding. Thus, unraveling genome-wide interspecific differences and genetic diversity can provide insights into the genetic mechanisms of growth and development of *G. uralensis*. Besides, it can also help in designing effective conservation and management strategies for *G. uralensis* (Hamrick et al., 1992; Hamrick and Godt, 1996). Based on the genetics of phenotypic variation, genome-wide information of licorice can be used in breeding to meet the increasing demand of breeders for desired traits while maintaining sufficient genetic variation to achieve continuous genetic gains over the breeding cycles.

In this study, leaf samples of *G. uralensis* species were collected from different geographical distributions in Xinjiang. Nutritional and photosynthetic indicators in leaves were evaluated to clarify the effects of geographical distribution on the growth of medicinal plants. Furthermore, whole-genome resequencing was used to explore the genetic diversity, genetic structure and adaptation mechanisms of *G. uralensis* populations at different geographical distributions. We hypothesized that the geographical position changed the nutrient and photosynthetic of the *G. uralensi*s leaves, and adjusted the genetic diversity and population adaptation of the *G. uralensis* population at the gene and population evolution level. The aim of this study was to (1) identify the geographical differences in nutrition and photosynthesis, (2) elucidate the geographical differences in genetic structure and genetic diversity of *G. uralensis* population, and (3) determine the evolution adaptation of *G. uralensis* population.

## Materials and Methods

### Specimen Collection

The experiments were conducted in Xinjiang Province, China, which has a wide distribution of *G. uralensis*. This research site was divided based on the geographical location as central (temperate continental arid semi-arid climate), southern (temperate continental arid climate), and eastern (temperate continental arid climate) regions (Supplementary Figure 1). The central and eastern regions of Xinjiang are separated from the southern region by the Tianshan Mountains, and the eastern and central regions are separated by the Turpan Basin. The average annual temperature in the central, southern, and eastern regions was 2–15, 5–18, and 3–17°C, respectively, the average annual precipitation was 180–270, 40.1–98.8, and 45.5–200 mm, respectively. The soil in the sampling area was all sandy soil. Field sampling was conducted in August 2019 to collect *G. uralensis* species leaf samples from different geographical locations. A total of twenty individual plants each from the east, central, and southern regions of Xinjiang Province, China, were collected from eight natural populations of *G. uralensis*. The collected leaf samples were immediately stored in liquid nitrogen for genomic DNA extraction. Concurrently, we determined nutrient elements and photosynthesis in the leaf from the same *G. uralensis* plants in each sampling area.

### Determination of Nutrient Elements and Photosynthesis in Leaves

The *G. uralensis* leaf samples collected from different areas were air-dried naturally indoors to a constant weight. Later, the dried leaf samples were ground into powder using a pestle and mortar. This powder was sieved using a 60-mesh sieve and used to determine nutrient levels in the leaves as described previously (Dang et al., 2020). Concisely, total nitrogen (PTN) was determined by perchloric acid-sulfuric acid digestion method, total phosphorus (PTP) by acid solution method (molybdenum antimony anti-colorimetric), total carbon content (POC) by potassium dichromate external heating method, and total potassium (PTK) content by acid digestion method (atomic absorption spectrometry).

In total, 0.5 cm diameter *G. uralensis* leaf sample was punched and extracted with 80% acetone in the dark at room temperature for 48 h (Li et al., 2004). The resulting green-colored supernatants were obtained after centrifuged at 5,000 rpm for 15 min, and the chlorophyll a (Chla), chlorophyll b (Chlb), and carotenoid content (Car) were measured using UV spectrophotometry at 664, 647, and 480 nm wavelength, respectively. The photosynthetic indicators in *G. uralensis* intact leaves with three regions [central (SW), eastern (UW), and southern (YW)] were all measured using a dual-PAM-100 device (Heinz Walz, Effeltrich, Germany) between 9: 00 and 12: 00, as described previously by Ritchie and Bunthawin (2010). The leaves were treated in darkness with dark adaptation clip for 25 min before the measurement. Later, chlorophyll fluorescence parameters, including Fv/Fm (photosystem II maximal photochemical efficiency), Y (II) (effective quantum yield of photosystem II), NPQ (non-photochemical quenching), Y (NO) (quantum yield of non-regulated energy dissipation), Y (NPQ) (quantum yield of regulated energy dissipation), ETR (I) (apparent rate of photosynthetic electron transport of photosystem I), and ETR (II) (apparent rate of photosynthetic electron transport of photosystem II), were recorded.

The analysis of variance (ANOVA) was used to analyze the effects of geographical location on the leaf nutrient elements, chlorophyll content, and chlorophyll fluorescence parameters in *G. uralensis*, and the statistically significant differences were performed by SPSS (version 19.0) (IBM Inc., Armonk, United States) followed by Bonferroni’s statistical test for multiple comparisons, and the significance level was set to 0.05.

### DNA Extraction and Genome Re-sequencing

The genomic DNA from all sixty leaf samples was extracted using a DNA secure Plant Kit (Tiangen, China), as per the manufacture’s instruction, and subsequently used to construct a sequencing library. Before library construction, NanoDrop2000 (Thermo Fisher Scientific, United States) was used to detect the concentration and purity of the extracted DNA at A260/A280 nm, and 1% agarose gel electrophoresis was used to determine DNA integrity. The qualified genomic DNA samples were cut into 300 to 500 bp fragments using the Covaris ultrasonic crushing instrument (Covaris ME220, Covaris, United States). These fragments were repaired and spliced through PCR amplification to construct the DNA library. Sequencing libraries were constructed with Invitrogen Collibri ES DNA Library Prep Kit (Set C, 49–72, Collibri*™*, Thermo Fisher Scientific). Paired-end sequencing with 150 bp read length was performed using an Illumina HiSeq 2500 sequencer based on the manufacturer’s instructions for genome sequencing of *G. uralensis* on the Illumina HiSeq 2500 platform (Illumina, San Diego, CA, United States) with 10× sequencing depth. Library size evaluation (insertion size of 150 bp) with micro tip electrophoresis with Agilent Bioanalyzer 2100 system and accurately quantification effective concentration (3 nmol/L) with q-PCR (Applied Biosystems*™* 7500, Thermo Fisher Scientific) are performed before Illumina sequencing to ensure the quality of the library.

### Sequencing Data Statistics, Quality Control, and Data Processing

Trimmomatic software (version 0.32) (Bolger et al., 2014) with the in-house script “unpairedOutFastqto taxon_filter.py trim” was used to delete the adapter sequences of the raw reads and then read the alignment (Lohse et al., 2012). The raw reads with more than 10% N content of the read length ratio and low-quality reads (the number of bases ≤ 10 accounted for more than 50% of the read length ratio) were eliminated. Clean data with high quality was obtained after strict filtering and quality control of raw reads. The BWA software (version 0.7.10) (Li et al., 2009) with the “MEM-t 4 -k 32 -M-R” parameter was used to align the high-quality sequencing data to the *G. uralensis* reference genome (Mochida et al., 2017)^1^, and after this analysis, duplicate reads were removed using SAMtools application with “rmdup” parameter (Li et al., 2009). In addition, SAMtools genotype likelihood model with the “doSaf” parameter was used to calculate genome-level DNA sequence polymorphisms caused due to single nucleotide variations or single nucleotide polymorphisms (SNPs), including single-base transitions, transversions, and so on. HaplotypeCaller method and GenotypeGVCFs modules in software GATK (version 3.7.1) (DePristo et al., 2011) were employed to perform SNP calls and variant detection of multiple samples. To improve the reliability and accuracy of SNP and genotype calls, QUAL < 30| | QD < 2.0| | MQ < 40.0| | FS > 60.0 Variant Filtration parameters were used to filter mutation sites. After filtration, high-quality SNP sites were obtained for subsequent analysis. ANNOVAR software^2^ (Yang and Wang, 2015) is a highly effective software tool, which uses updated information to annotate the genetic variation in multiple genomes (San Lucas et al., 2012). Due to the efficient annotation function and wide recognition of ANNOVAR in the scientific community, we used it to annotate the filtered high-quality SNPs. The genome re-sequencing data used in this study have been deposited in the Sequence Read Archive (SRA) database of National Center for Biotechnology Information (NCBI) (PRJNA 730103)^3^.

### Population Structure and Genetic Diversity Analysis

A phylogenetic tree describes the evolutionary relationships between different populations. The SNPs obtained in this experiment were used to calculate the distance between populations using a distance matrix by employing TreeBest^4^ software. For *G. uralensis* samples from different geographical regions, the neighbor-joining method was employed to construct a phylogenetic tree with bootstrap values calculated 1000 times. Principal component analysis (PCA) based on the calculation of SNPs differences in individual genomes was used to cluster individuals into different subgroups according to principal components of different traits. In genetics, PCA is mainly used for clustering analysis. In this study, based on the SNP differences between individuals, GCTA software^5^ was used to calculate the eigenvectors and eigenvalues. R software^6^ was employed to construct the PCA distribution map. Population genetic structure refers to a non-random distribution of genetic variation within a species or population. A population can be divided into several subgroups according to the criteria of geographical distribution. Individuals within the same subgroup are closely related to each other, but the relationships between different subgroups are slightly distant. The analysis of population structure could unravel the evolutionary process. PLINK software and structure software (Pritchard et al., 2000) were used to construct population lineage information and population genetic structure based on the rate of change in the log probability of data between consecutive K values (Evanno et al., 2005).

Based on the filtered SNP loci, the genetic diversity index of each population was calculated using Arlequin software (Excoffier et al., 2005) with the “dp4-miss0.1-Maf0.05” parameter. Among these genetic diversity indicators, the observed homozygosity (Ho) and expected heterozygosity (He) reflect the degree of genetic variation in the population. Higher heterozygosity represents higher genetic diversity within the population. Nucleotide diversity (π) is the heterozygosity at the nucleotide level, reflecting the level of polymorphism in the population. Analysis of Molecular Variance (AMOVA) refers to the analysis of molecular differences, which can unravel corresponding genetic variation among different geographic groups.

### Linkage Disequilibrium Analysis

Linkage disequilibrium (LD) refers to the non-random association between alleles at different loci within a population. In LD, the probability of coexistence of two alleles on the same chromosome is greater than the probability of co-occurrence due to random distribution in the population. LD is denoted by its parameter (R^2^). LD analysis was performed using VCFtools (version 0.1.12b) (Danecek et al., 2011), which evaluated the LD value over each 10 kbp window and calculated the R^2^ between SNPs with pairwise distances larger than 1 kbp.

### Historical Dynamic Analysis of Populations

Effective population size (Ne) represents a population with the same gene frequency variance as the actual population, reflecting the average homozygous rate of genes in the population genetic structure. Ne in *G. uralensis* population from different geographical locations was evaluated through the pairwise sequentially markovian coalescent (PSMC) method (Li and Durbin, 2011) after determining the LD between SNPs. As a single plot could not accommodate all the groups, we selected five representative individuals from each group to construct the PSMC plot.

### Selective Elimination Analysis

The fixed coefficient F of the population, which is a special case of the F statistic (Fst), reflects the level of allelic heterozygosity of the population. Fst was calculated using VCFtools software (Danecek et al., 2011) with a non-overlapping window size of 10 kbp and step size of 20 kb. Frequency profiles were constructed based on Fst with high-quality SNP loci for selective elimination analysis. Fst and θπ are highly effective methods for determining the selection elimination region, specifically during mining the functional region closely related to the survival environment, which often results in a strong selection signal. In this study, Fst and θπ were used to perform selection elimination analysis between populations, which could jointly screen strong selection signals and facilitate the screening of target genes.

### Gene Functional Enrichment Analysis

The candidate genes in selected regions obtained after selective elimination analysis were subjected to the Gene Ontology (GO) functional analysis and Kyoto Encyclopedia of Genes and Genomes (KEGG) pathway analysis. In GO analysis, all candidate genes were queried in the GO database^7^, and the number of target genes mapping to each term was calculated. The *P*-values obtained through hypergeometric test and corrected using Bonferroni several times (threshold value set ≤ 0.05) were applied to identify significantly enriched GO terms for the candidate target genes (Du et al., 2010). The biological functions of the candidate target genes were determined by the significant enrichment analysis of GO function.

Kyoto Encyclopedia of Genes and Genomes is an open-access database of signal transduction pathways. A hypergeometric test was used in KEGG pathway enrichment analysis to determine significantly enriched pathways for the candidate genes compared to the entire reference gene (*p*-value less than 0.05 after correction) (Mao et al., 2005). The important biochemical metabolic pathways and signal transduction pathways for the candidate genes were determined by pathway significance enrichment analysis.

## Results

### Effects of Geographical Location on *G. uralensis* Leave Nutrient Element Levels

Analysis of variance results demonstrated significant differences in levels of carbon (POC), nitrogen (PTN), phosphorus (PTP), potassium (PTK), and other stoichiometric indices in *G. uralensis* leaf samples from central (SW), eastern (UW), and southern (YW) regions of Xinjiang (Table 1). The POC levels in *G. uralensis* leaf samples from the central (SW) were significantly higher than in the southern (YW) (*p* < 0.05). In contrast, the PTP levels in *G. uralensis* leaf samples from the southern (YW) were significantly higher than in central (SW) and eastern (UW) (*p* < 0.05). There was no significant difference in PTN and PTK levels between the three regions (*p* > 0.05). In addition, C: N, C: P, and N: P ratios differed significantly in *G. uralensis* leaf samples from different geographical locations (*p* < 0.05). Specifically, the ratio of C: N in the *G. uralensis* leaf samples from the central (SW) was significantly higher than in the eastern (UW) (*p* < 0.05). However, the C: P and N: P ratios in the southern (YW) were significantly lower than in the central (SW) and eastern (UW) (*p* < 0.05) (Table 1).

**TABLE 1:** Effects of geographical location on leaf nutrient elements in *G. uralensis*.

	POC (g/kg)	PTN (g/kg)	PTP (g/kg)	PTK (g/kg)	C:N	C:P	N:P
UW	412.617 ± 2.304 ab	24.886 ± 1.110 a	1.274 ± 0.011 b	12.330 ± 0.488 a	16.637 ± 0.633 b	323.918 ± 4.530 a	19.546 ± 1.008 a
SW	438.943 ± 9.638 a	20.392 ± 1.125 a	1.193 ± 0.019 b	8.865 ± 0.576 a	21.617 ± 0.868 a	368.141 ± 10.802 a	17.087 ± 0.843 a
YW	391.523 ± 7.810 b	20.981 ± 0.576 a	1.816 ± 0.144 a	13.170 ± 1.601 a	18.672 ± 0.279 ab	218.784 ± 20.945 b	11.703 ± 1.023 b

*UW, SW, YW represents G. uralensis leaves of eastern, central, and southern regions, respectively. POC, leaf total carbon content; PTN, leaf total nitrogen; PTP, leaf total phosphorus; PTK, leaf total potassium; C:N, the ratio of carbon and nitrogen; C:P, the ratio of carbon and phosphorus; N:P, the ratio of nitrogen and phosphorus. Value is Mean ± SE, different lower-case letters represented a significant difference (p < 0.05) was assessed by one-way analysis of variance followed by Bonferroni’s statistic test for multiple comparisons, the same letter indicates no significant difference (p > 0.05).*

### Effects of Geographical Locations on Photosynthesis Indices of *G. uralensis* Leaves

**FIGURE 1:**
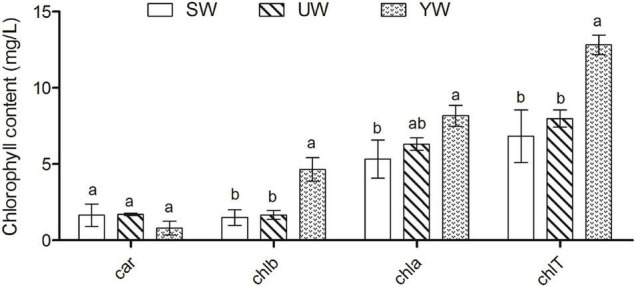
Effects of geographical location on leaf chlorophyll content in *G. uralensis*. Bar charts (mean with standard error) with different lower-case letters represented a significant difference (*p* < 0.05) was assessed by one-way analysis of variance followed by Bonferroni’s statistic test for multiple comparisons. The same letter indicates no significant difference (*p* > 0.05). Car, carotenoid contents; chlb, chlorophyll b; chla, chlorophyll a; chlT, total chlorophyll.

The highest Chla levels were found in *G. uralensis* leaves samples from the southern (YW), and chlorophyll a (Chla) levels were significantly higher in *G. uralensis* leaf samples from the central (SW) based on ANOVA outcomes (*p* < 0.05). The chlorophyll b (Chlb) and total chlorophyll (ChlT) levels in the *G. uralensis* leaves samples from the southern (YW) were the highest than that in eastern (UW) and central (SW) (*p* < 0.05). However, carotenoid contents (Car) did not differ significantly between the three regions (*p* > 0.05) (Figure 1).

Based on ANOVA, geographical location significantly affected the *G. uralensis* chlorophyll fluorescence (*p* < 0.05) (Supplementary Table 1). Specifically, ETR (I) and ETR (II) in *G. uralensis* leaf samples from southern (YW) were significantly higher than in central (SW) and eastern (UW) samples (*p* < 0.05), but the geographical location did not implicate significant differences in Fv/Fm, Y (II), NPQ, Y (NO),Y (NPQ) indices (*p* > 0.05) (Supplementary Table 1).

### Whole-Genome Re-sequencing of *G. uralensis*

Total 150.59 Gb was obtained in the whole-genome re-sequencing of sixty *G. uralensis* leaf samples. Genome coverage was 6.27× through sixty individuals. After subjecting these data to strict filtering, 150.06 Gb high- quality clean data was obtained. The sequencing data output statistics for each sample are listed in Supplementary Table 2. Q20 ≥ 95 and Q30 ≥ 89% indicate high sequencing quality, normal GC distribution (36.38–39.32%, with an average of 37.6%), 99.65% effective rate, and 0.03% error rate of data.

Based on BWA software, 895237686 reads from 997491918 valid sequencing reads were aligned against the chromosome level high-precision genome (Mochida et al., 2017). Supplementary Table 3 demonstrated the sequencing depth and coverage of each sample in detail. The mapping rate ranged from 80.62 to 91.93% (average 89.7%), reflecting the similarity between the sample’s sequencing data and the reference genome. The coverage depth and coverage reflect the uniformity and homology of the sequencing data with the reference sequence. The average sequencing depth of samples was 9x. The average sample coverage was 87.02% (coverage of at least one base). Sample comparison outcomes demonstrated that the similarity of the samples with the reference genome met the requirements of re-sequencing analysis.

### Single Nucleotide Polymorphism Detection and Annotation

SAMtools software was used to detect SNPs in the *G. uralensis* sequencing data. A total number of 6985987 SNPs were identified. After filtering, 117970 high-quality SNPs were obtained for subsequent analysis. ANNOVAR software annotation results showed that most SNPs were located in the intergenic region (84.74%), and only 2.75% of SNPs were located in the exonic region. In the exonic region, 1232 synonymous (amino acids in the coding region are not changed) and 1902 non-synonymous (amino acids in the coding region changed) transformations were observed. The ratio of non-synonymous to synonymous transformations was found to be 1.54 (Table 2).

**TABLE 2:** SNPs distribution in *G. uralensis* species.

Category		Number of SNPs
Exonic	Upstream	2858
	Stop gain	107
	Stop loss	5
	Synonymous	1232
	Non-synonymous	1902
Intronic		8850
Splicing		38
Downstream		2251
upstream/downstream		336
Intergenic		99972
ts		86134
tv		31836
ts/tv		2.705
Total		117970

*Exonic refers to the regions where the mutation is located in the exon; Upstream refers to the 1 Kb region of upstream gene; Stop gain refers to a mutation that makes a gene acquire a stop codon; Stop loss refers to a mutation that deprives a gene of its stop codon; Synonymous refers to a synonymous change; Non-synonymous refers to a variation that is not synonymous; Upstream, Stop gain, Stop loss, Synonymous and Non-synonymous are all in the Exonic region. Intronic refers to the region where the mutation is located in the intron region; Splicing is variation at the Splicing site (The intron is close to the exon/2 bp of the intron boundary); Downstream means the area 1 Kb Downstream of the gene; Upstream/Downstream refers to the Upstream 1 Kb region of a gene and will start on the Downstream 1 Kb region of another gene; Intergenic means that the variation lies between genes; transition (ts) refers to base transition; transversion (tv) refers to base transversion; ts/tv refers to the ratio of conversion to transversion; Total refers to the total number of SNPs.*

### Effects of Geographical Location on Population Structure and Genetic Diversity of *G. uralensis*

**FIGURE 2:**
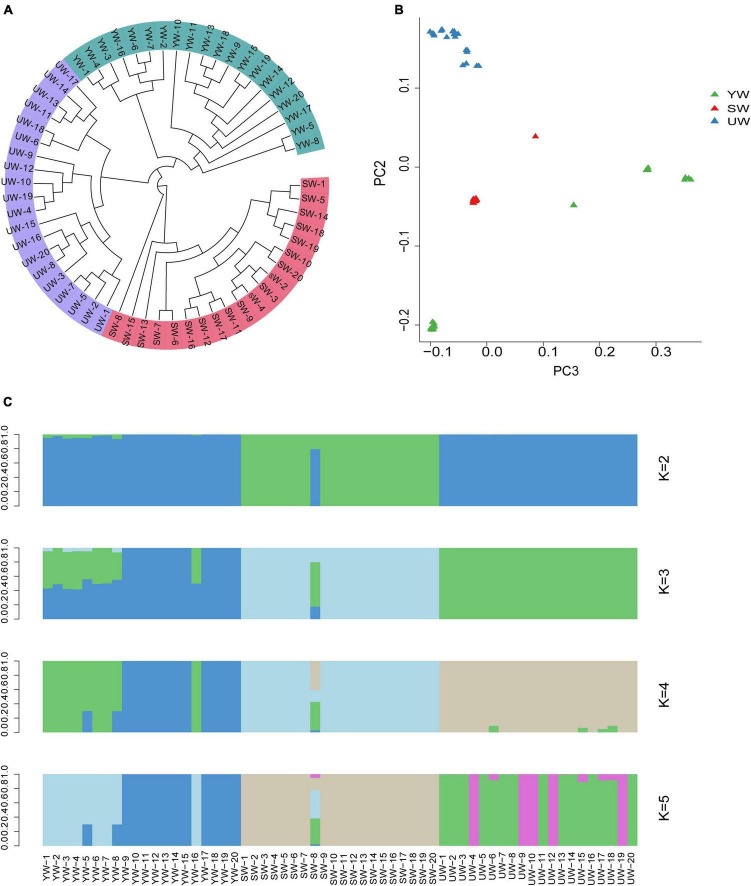
Population structure analysis based on individual SNPs. **(A)** Phylogenetic tree showing the evolutionary relationships between different populations, the evolutionary branches of more closely related population are grouped together and marked by the same color. **(B)** Principal component analysis (PCA), which each point in the diagram represents a sample, and samples from the same group are represented in the same color. The closer the distance between the samples, indicates the smaller the genetic background differences between the samples. And vice versa. **(C)** Population structure analysis, which *K* values represent the number of different subgroups, and different colors represent different subgroups. Ordinate is the proportion of different subgroups; abscissa is the sample name (UW, SW, YW refers to *G. uralensis* of eastern, central, and southern regions, respectively, number represents the replicate number).

A phylogenetic tree was constructed based on individual SNPs to evaluate the population stratification and genetic correlation between *G. uralensis* at a genome level. The outcomes demonstrated that the *G. uralensis* in the Xinjiang region could be categorized into three major clades: central (SW), eastern (UW), and southern (YW), reflecting strong genetic differentiation. This might be the outcome of long-term geographical isolation (Figure 2A). In addition, PCA showed that sixty samples analyzed in this study could be divided into three clusters based on the difference in the degree of SNPs in the individual genomes. Besides, the eastern (UW) population was separately clustered. The southern (YW) and the central (SW) samples were relatively dispersed compared to eastern (UW) samples (Figure 2B). It indicated differences in genetic distance between individuals in the *G. uralensis* population. These outcomes were further supported by population structure analysis performed using the Structure software. According to this analysis, when *K* = 3, the medicinal *G. uralensis* could be clearly divided into three populations: central (SW), eastern (UW), and southern (YW). When *K* = 4 or *K* = 5, the *G. uralensis* population could still be clearly divided into three different populations despite the presence of other colors or other genetic structures being evident (Figure 2C). When K value was minimum, two colors were evident in the central (SW) and southern (YW) population, but only one color was present for the eastern (UW) population. It indicated infiltration of the *G. uralensis* gene from the eastern (UW) to the southern (YW) and central (SW) population. These results suggest that *G. uralensis* populations from different regions may share a common origin, despite the genetic differences between populations.

To further evaluate the genetic diversity of *G. uralensis* populations in the Xinjiang region, we performed Ho, He, AMOVA, and π analysis. Arlequin software was employed to calculate the genetic diversity index of each population based on the filtered SNP loci. The outcomes showed that the values of Ho (0.492), He (0.324), and π (0.329) were the highest in the central (SW) population, and the values of Ho (0.436), He (0.3), and π (0.304) were the lowest in southern (YW) population (Supplementary Table 4). These outcomes indicated that the genetic diversity of *G. uralensis* populations varied according to the regions. Also, the genetic and nucleotide diversity in central (SW) populations was higher than that in southern (YW) populations. Molecular variance analysis demonstrated 8.72% of the significant genetic differences in *G. uralensis* samples from the population in the three regions and 149.97% of the significant genetic differences within individuals between different populations (*p* < 0.05) (Table 3). It indicated that the individual genetic differences were significant in each *G. uralensis* population.

**TABLE 3:** *G. uralensis* population analysis of molecular variance.

Source of variation	Degree of freedom (df)	Sum of squares	Variance components	Percentage of variation	*P*-value
Among populations	2	13417.917	153.401	8.72	0.064
Among individuals	57	32655.475	−1031.953	−58.69	0.056
Within individuals	60	158208.5	2636.808	149.97	**0.012**
Total	119	204281.892	1758.257		

*p < 0.05 indicates statistical significance. Bold values are less than 0.05, indicating statistical significance.*

### Population History and Effective Population Size Analysis

**FIGURE 3:**
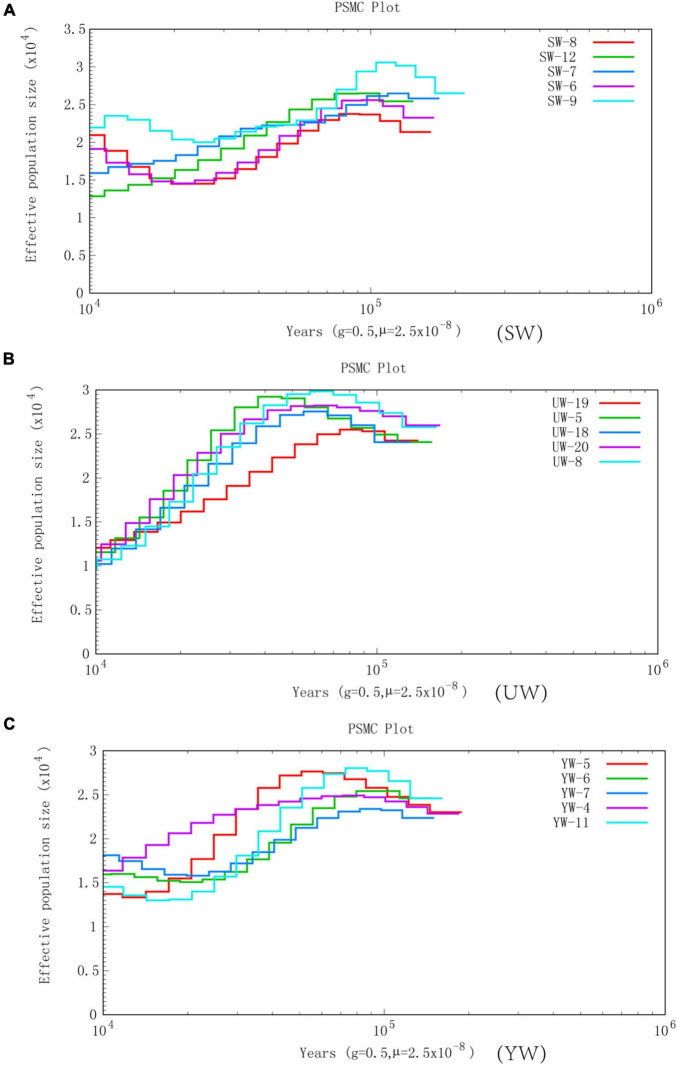
Effective population size is based on PSMC analysis. Abscissa represents different historical periods, ordinate represents the effective population size. In the figure, the generation period is 0.5 years (half a year), and the mutation rate of each generation is 2.5 × 10^–8^. **(A–C)** Represents *G. uralensis* of central, eastern, and southern regions, respectively.

In this study, we selected five representative individuals from each population to conduct population history and Ne analysis to determine the increment in average inbreeding number and average homozygous rate of genes in the genetic structure of the population. PSMC was used to reconstruct the evolutionary history of the *G. uralensis* population in three regions, revealing the fluctuation of *G. uralensis* effective population size from 10,000 to 100,000 years ago (Figure 3). PSMC simulation results showed relatively consistent fluctuations in the effective population size of the three *G. uralensis* populations. However, the effective population changed notably between 20,000 and 100,000 years ago. Specifically, the effective population size of licorice peaked around 100,000 years ago and then declined to bottleneck until about 15,000 years ago. Also, the effective population size began to increase about 15,000 years ago (Figure 3).

### Effects of Geographic Location on Linkage Disequilibrium

**FIGURE 4:**
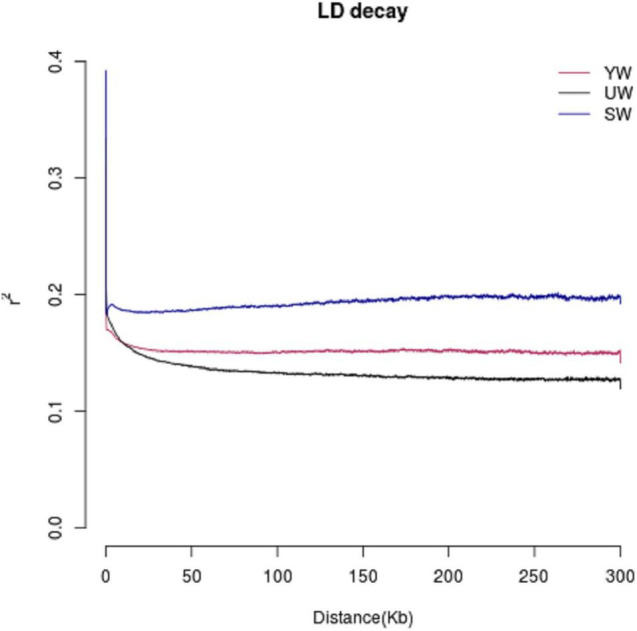
Linkage disequilibrium (LD) analysis of different *G. uralensis* populations. The abscissa indicates the distance at which linkage disequilibrium occurs, ordinate is linkage disequilibrium coefficient.

The LD value is represented in terms of linkage disequilibrium coefficient (R^2^). Higher R^2^ represents a stronger bond and a smaller corresponding SNP spacing. When R^2^ decays to half, the value of the LD decay is represented by the corresponding SNP interval. R^2^ values were found to be low (0.1–0.2) for central (SW), eastern (UW), and southern (YW) populations (Figure 4). It indicated that the three populations were not affected by random drift in a long period to reach higher frequencies. In addition, *G. uralensis* population from central, southern, and eastern regions showed different LD decay curves, indicating that the population demographic histories of the three regions were different, with the eastern (UW) population having the slowest decay rate and the central (SW) population having the fastest decay rate.

### Selective Sweep Analysis

To estimate population differentiation and screen for differential genetic regions between the two different groups, Fst statistics were calculated based on genome-wide SNP data to reflect the level of allelic heterozygosity in the population. A low Fst value (0.304 > Fst > 0.232) was obtained in the population differentiation analysis (Supplementary Figure 2), indicating that the three different *G. uralensis* populations in this study were not affected by genetic drift and migration.

**FIGURE 5:**
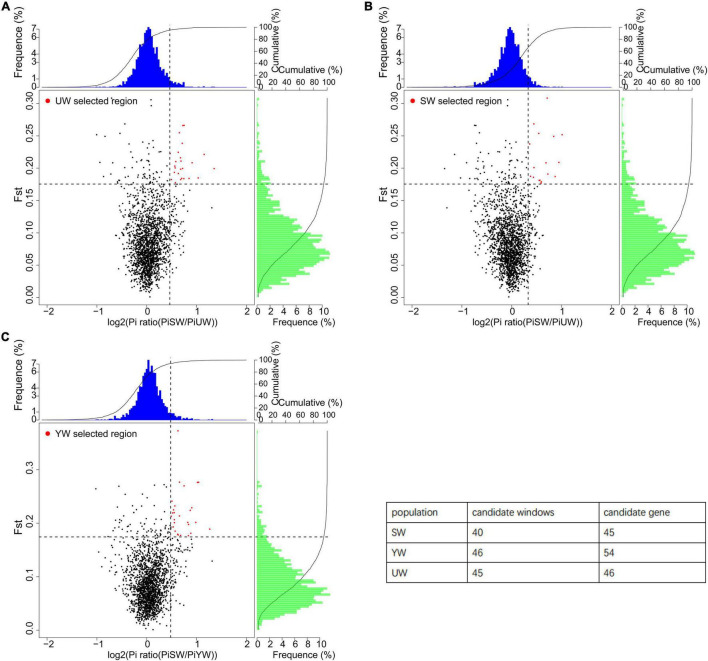
Selective sweep analysis of different *G. uralensis* populations. The abscissa is the θπ ratio value, which corresponds to the frequency distribution at the top of the figure. The ordinate is the Fst value, which corresponds to the frequency distribution on the right side of the figure. The points in the middle of the figure represent the corresponding values of Fst and θπ ratio in different Windows. The blue and green regions are the top 5% regions selected by θπ, and the red regions are the top 5% regions selected by Fst. **(A–C)** Represents *G. uralensis* of eastern, central and southern regions, respectively.

In this study, a total of 131 candidate regions were screened, and 145 candidate genes were identified using selective sweep analysis based on Fst and θπ (Figure 5). Specifically, regions with significantly higher Fst (Fst > 0.207) and log2 (PiP/PiH) > 0.716 were selected. Strong selection signals were screened out a total of 45 selected regions and 46 candidate genes from the eastern (UW) *G. uralensis* population (Figure 5A). Regions with significantly higher Fst (Fst > 0.211) and log2 (PiP/PiH) > 0.623 were selected. Strong selection signals were screened out a total of 40 selected regions and 45 candidate genes from the central (SW) *G. uralensis* population (Figure 5B). Regions with significantly higher Fst (Fst > 0. 0.221) and log2 (PiP/PiH) > 0.705 were selected. Strong selection signals were screened out a total of 46 selected regions and 54 candidate genes from the southern (YW) *G. uralensis* population (Figure 5C).

### Gene Functional Enrichment Analysis

**FIGURE 6:**
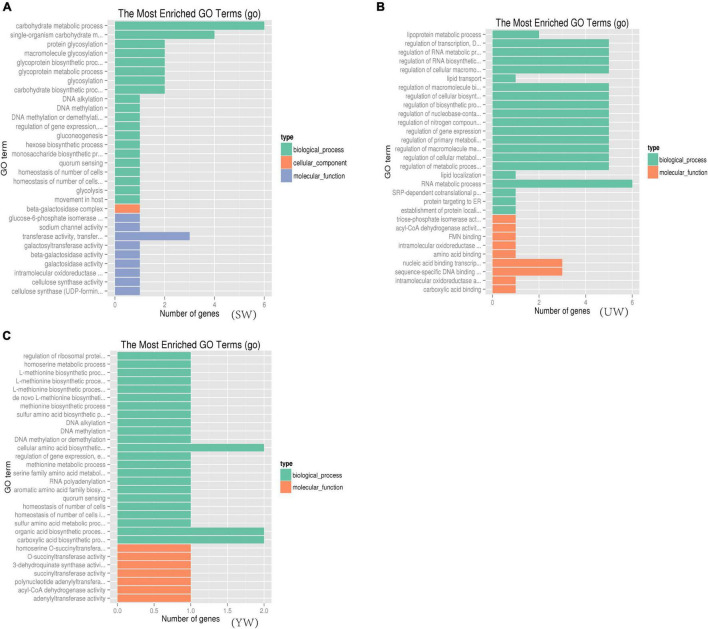
Gene ontology (GO) functional significance enrichment analysis based on candidate gene. **(A–C)** Represents *G. uralensis* of central, eastern and southern regions, respectively.

To understand the biological function of candidate genes (such as Glyur001802s00036258, Glyur003702s00044485, Glyur001802s00036257, Glyur007364s00047495, Glyur0000 28s00003476 and Glyur000398s00034457) identified in the selected regions, Gene Ontology (GO) and Kyoto Encyclopedia of Genes and Genomes (KEGG) analyses were performed for their functional enrichment. A total of 690 GO terms [eastern (UW) = 214, central (SW) = 220, southern (YW) = 256] were mapped, among them 110 GO terms were significantly enriched (*p* < 0.05) in the GO functional analysis of *G. uralensis* populations from three distinct geographical regions. The GO functional categories enriched included 76 biological processes, 32 molecular functions, and 2 cellular components (Supplementary Table 5). Figure 6 showed the 30 GO terms with the greatest abundance in the GO functional analysis in the *G. uralensis* species. As shown in Figure 6, multiple significantly enriched GO terms were related to carbohydrate metabolism and biosynthesis *p* < 0.05), including carbohydrate metabolic process (GO: 0005975, GO: 0009100, GO: 0070085), carbohydrate biosynthetic process (GO: 0016051), glucose catabolic process (GO: 0006007), carbohydrate derivative biosynthetic process (GO: 1901137), monosaccharide biosynthetic process (GO: 0046364), hexose catabolic process (GO: 0019320), single-organism carbohydrate metabolic process (GO: 0044723), hexose biosynthetic process (GO: 0019319), protein glycosylation (GO: 0006486), glycoprotein biosynthetic process (GO: 0009101), galactosyltransferase activity (GO: 0008378), beta-galactosidase complex (GO: 0009341), gluconeogenesis (GO: 0006094), beta-galactosidase activity (GO: 0004565), galactosidase activity (GO: 0015925), glycolysis (GO: 0006096) (Figure 6). In addition, triose-phosphate isomerase activity (GO: 0004807), lipoprotein metabolic process (GO: 0042157), acyl-CoA dehydrogenase activity (GO: 0003995), lipid transport (GO: 0006869), mitochondrial electron transport, NADH to ubiquinone (GO: 0006120), mitochondrial ATP synthesis coupled electron transport (GO: 0042775), which were closely related to the tricarboxylic acid cycle, were also significantly enriched (*p* < 0.05) (Figure 6).

To unravel the signaling pathways associated with the candidate genes in the three populations, KEGG pathway analysis was performed on the selected candidate genes. A total of 17 KEGG pathways [eastern (UW) = 9, central (SW) = 1, southern (YW) = 7] were enriched in the three populations, of which 7 KEGG pathways [eastern (UW) = 3, central (SW) = 1, southern (YW) = 3] were significantly enriched (*p* < 0.05) (Table 4). As shown in Table 4, alpha-linolenic acid metabolism (ath00592), Biosynthesis of unsaturated fatty acids (ath01040) and fatty acid degradation (ath00071) pathways were significantly enriched in both eastern (UW) and southern (YW) populations (*p* < 0.05). In the central (SW) population, only the spliceosome (ath03040) signaling pathway was significantly enriched (*p* < 0.05). These results indicate that functional genes related to biochemical metabolic and signal transduction pathways play a crucial role in the population differentiation of *G. uralensis* from central, southern, and northeastern regions.

**TABLE 4:** *G. uralensis* population pathway analysis of Kyoto Encyclopedia of Genes and Genomes (KEGG) based on candidate gene.

Population	Term	Gene number	Background number	*P*-value	Corrected *P*-value	Gene name
**UW**	alpha-Linolenic acid metabolism	2	33	**0.001**	0.005	Glyur000178s00013231| Glyur000345s00015647
	Biosynthesis of unsaturated fatty acids	1	36	**0.038**	0.128	Glyur000345s00015647
	Fatty acid degradation	1	40	**0.043**	0.128	Glyur000345s00015647
	Fatty acid metabolism	1	73	0.076	0.150	Glyur000345s00015647
	Peroxisome	1	81	0.083	0.150	Glyur000345s00015647
	Ribosome biogenesis in eukaryotes	1	101	0.103	0.154	Glyur002421s00038665
	Spliceosome	1	192	0.186	0.240	Glyur002421s00038663
	Ribosome	1	360	0.322	0.363	Glyur000016s00003034
	Metabolic pathways	2	1861	0.613	0.613	Glyur000178s00013231| Glyur000345s00015647
SW	Spliceosome	1	192	**0.019**	0.019	Glyur000030s00002364
YW	alpha-Linolenic acid metabolism	1	33	**0.026**	0.073	Glyur000345s00015647
	Biosynthesis of unsaturated fatty acids	1	36	**0.028**	0.073	Glyur000345s00015647
	Fatty acid degradation	1	40	**0.031**	0.073	Glyur000345s00015647
	Fatty acid metabolism	1	73	0.056	0.086	Glyur000345s00015647
	Peroxisome	1	81	0.061	0.086	Glyur000345s00015647
	Oxidative phosphorylation	1	162	0.119	0.139	Glyur000133s00010348
	Metabolic pathways	2	1861	0.434	0.434	Glyur000345s00015647| Glyur000133s00010348

*UW, SW, YW refers to G. uralensis of eastern, central, and southern regions, respectively. Bold values are less than 0.05, indicating statistical significance.*

## Discussion

In this study, sixty *G. uralensis* samples were subjected to whole-genome re-sequencing to explore the evolutionary patterns and genomic differentiation in *G. uralensis* from different geographical locations. After filtering out the low-quality readings, a total of 150.06 Gb of high-quality re-sequenced clean reads and 117970 SNPs were obtained. The mutation sites in gene regions significantly impact the growth and development, stress, and synthesis of bioactive components of *G. uralensis* plants (Xanthopoulou et al., 2019; Wang et al., 2020). Nasrollahi et al. (2014) reported that squalene synthase (SQS) and β-amyrin synthase (bAS) upregulation conferred drought stress resistance to *Glycyrrhiza glabra* seedling and adult plants. He et al. (2021) reported that the cytochrome P450 monooxygenase (CYP450) gene (Cluster-30944.70498) up-regulation was positively correlated to the bioactive component of licorice (glycyrrhizic acid). Gong et al. (2015) demonstrated that *G. uralensis* extract could induce expression of nuclear factor erythroid 2-related factor 2 (Nrf2) and its downstream genes that play a vital role in protection against toxic xenobiotics. In this study, up to 84.7% of SNPs were in the intergenic regions, 1902 SNPs in the non-synonymous regions, and 2858 and 2251 SNPs were determined to be located in the upstream and downstream regions, respectively. Thus, *G. uralensis* species may have diverse phenotypes and ecological adaptations. One plausible reason might be that *G. uralensis* population from widely distributed habitats (both temperate grassland and desert areas) with differences in drought levels, light and heat resources and salinity (Zhang et al., 2005) might have a broad geographic origin, which might have promoted more adaptive mutations. The data obtained in this study provide a theoretical basis for genetic improvement of *G. uralensis* plant.

### Genomic Differentiation of *G. uralensis* Population

In this study, we used whole-genome re-sequencing techniques to investigate the genetic structure, population evolutionary history, and genomic differentiation of *G. uralensis* from different regions. *G. uralensis* can be roughly categorized into three groups based on their ecological characteristics and geographical distribution: central, eastern, and southern (Figure 2) populations. Phylogenetic analysis and PCA of *G. uralensis* from these regions showed clear genetic differentiation among the three populations. The complex geographical conditions in Xinjiang resulted in long-term geographic isolation between these three populations, and we speculated that geographical barriers might cause population differentiation and a discontinuous distribution pattern between different *G. uralensis* populations from Xinjiang, China. According to the previous studies (Qiu et al., 2011), historical geological changes and climatic oscillations can result in geographical barriers that affect the geographical distribution patterns of multiple plant species, resulting in population differentiation. Climatic fluctuations and regional uplift (on the Tibetan Plateau) during the quaternary period (about 2.6 million years ago) led to geographic isolation between different populations, including the emergence of glacial refuges. This, in turn, disrupted the distribution patterns of plants and fragmented their ranges (Du et al., 2015).

The last glacial age occurred between 0.11 and 0.02 Ma (Mega annum), which witnessed dramatic climatic fluctuations and historic tectonic changes. It led to population differentiation and even the extinction of multiple plant species in northern China (Cheng et al., 2005; Shen et al., 2005; Zhang et al., 2005). In our study also, we observed significant genetic differences in *G. uralensis* plants from the eastern, central, and southern regions, which supports the geographic barrier hypothesis.

Furthermore, genetic differentiation among populations is strongly influenced by genetic drift and gene flow (Hamrick and Godt, 1996). Analysis of population structure in this study showed that when *K* = 3, only one color was present for the eastern (UW) population, but it did not exclude possible hybridization in other regions (Figure 2C), indicating gene flow in the genetic structure of *G. uralensis* population. We speculate that the dispersal of *G. uralensi*s seeds through grazing activities (cattle and sheep), human development activities, and animals (birds) could be considered as the primary factors for the gene flow.

In this study, we explored the *G. uralensis* genetic diversity and observed that the Ho and He for *G. uralensis* ranged between 0.492–0.436 and 0.324–0.3, respectively (Supplementary Table 4). These Ho and He values were relatively low compared to other Chinese herbal medicines, including *Epimedium* (Zhou et al., 2010), *Himatanthus drasticus* (Baldauf et al., 2011), and *Docynia delavayi* (Peng et al., 2021). According to previous reports, species with a narrow or endemic distribution reportedly maintained lower genetic diversity than those with a broader distribution (Hamrick and Godt, 1996), which is in line with the current study. It suggests that the *G. uralensis* population habitat is restricted to a small range, which may be associated with high habitat disturbance and small population size.

In addition, we observed that the genetic diversity of *G. uralensis* populations in the central (SW) region was slightly higher than that in southern (YW) and eastern (UW) regions (Supplementary Table 4). This might result from their distribution under complex geographical and climatic conditions due to more robust natural selection (Zhao and Gong, 2015). We believe that this may be related to the biological characteristics of the species, and the age of *G. uralensis* samples used is a hidden variable factor. In general, long-lived species have higher levels of genetic variation within populations and lower levels of genetic differentiation between different populations, reflecting high levels of gene flow (Hamrick and Godt, 1996; Finkeldey and Hattemer, 2007; Brütting et al., 2012). The difference in the adaptability of the different populations to the environment results in the eventual accumulation of genetic variation in the populations, resulting in the different degrees of genetic differentiation between different populations (Charlesworth et al., 1995). Patterns of genetic variation within species and between populations result from evolutionary processes and other historical factors. High genetic diversity helps a particular species to adapt to its natural habitat (Hittinger et al., 2010). The high genetic diversity of the *G. uralensis* population in the central region may indicate better ecological adaptability and dependence on habitat environment.

In addition, we found that the leaf nutrient content (POC and PTP), chlorophyll content (Chla, Chlb and ChlT) and chlorophyll fluorescence parameters (ETR (I) and ETR (II) of *G. uralensis* population were significantly different in the three regions (*p* < 0.05) (Figure 1 and Supplementary Table 1). This suggests that different populations adapted differently to the local area (light, temperature, humidity and soil characteristics) at the physiological level (Gaastra, 1959; Incropera, 1975). This is related to the impact of environmental factors on plant growth, and how plants respond to environmental heterogeneity or stress by regulating their growth level, which may be manifestations of a tradeoff strategy for plant survival in the natural environment. However, the main limitation of the current research results is that leaves samples of *G. uralensis* plants were collected from different geographical regions to prove the influence of complex geographical conditions on plant nutrients and photosynthesis, and lack of information about differences at the physiological level at common garden experiment or the same condition. Although further research is needed to characterize differentiated phenotypes genetically in this species, our study provides useful information about the effect of geographical conditions on plant phenotypic differentiation.

### Evolution of *G. uralensis*

Genomic data contains vast information on the evolutionary loci. We evaluated the genetic parameters to infer the origin and evolution of *G. uralensis* population in Xinjiang, China. Based on the genomic information, we observed higher Ho, He, and π values of *G. uralensis* population in the central region than in the southern and eastern populations (Supplementary Table 4). It indicated the highest genetic diversity in the central and lowest genetic diversity in the southern *G. uralensis* population. According to a study by Zhao et al. (2013), the exceptionally low genetic diversity of Tibetan barley species in Tibet indicates that Tibet is unlikely to be the origin or domestication center of barley. Similarly, we can infer that the central region is the center of origin for *G. uralensis* and not the southern region of Xinjiang, China. Numerous species have experienced strong positive selection in the long course of evolution. These selected regions exhibited selective elimination of signal and resulted in a decrease in SNP and an increase in LD. Due to the initiation of selection, domesticated populations mostly have higher LDs than wild populations, and therefore, species with faster LD decay are more primitive (Guo et al., 2011; Xia et al., 2015). According to the LD analysis in this study, the linkage probability between SNPs in the central population was relatively high, with the fastest rate of decay (Figure 4). This validated our hypothesis that *G. uralensis* might have originated in the central region of Xinjiang, China. In conclusion, the central population had the highest genetic diversity, nucleotide diversity, and allele frequency, indicating that the central region was the *G. uralensis*’ center of origin in Xinjiang. The migration routes of licorice in central, northeast, and southern regions had an “inverted C” migration model. *G. uralensis* is one of the most widely distributed and ecologically important medicinal herbs in Xinjiang, China. The study on the origin and migration pathway of *G. uralensis* holds a vital reference value, which will further our understanding of the geographical distribution pattern of other medicinal plant populations.

In this study, genomic sites of the entire *G. uralensis* population had lower π and higher Fst values compared to candidate sites (Supplementary Figure 2 and Supplementary Table 4). Specifically, the absence of gene flow, selection due to local ecological adaptation, and other reasons could result in the decreased diversity within the population and indirectly inflate the Fst values (Cruickshank and Hahn, 2014). In positive selection, genetic diversity decreased, and interspecific differentiation increased. Under the selective scanning model, gene variants associated with beneficial mutations of positive selection hitched and reached higher frequencies (Kaplan et al., 1989). As the main evolutionary force, positive selection is closely correlated to genomic differentiation of the *G. uralensis* population; however, background selection cannot be completely ignored. Since it is difficult to accurately estimate the variation in these regions that exhibit low genetic diversity, the functional characterization of these genes must be performed cautiously. Thus, further studies on these functional genes are required to elucidate the balanced selection in the process of adaptive evolution of the *G. uralensis* population.

In this study, 131 candidate regions were screened, and 145 candidate genes were identified based on selective elimination analysis (Figure 5). This provided information for further study on genetic differentiation and environmental adaptation of the *G. uralensis* population. In GO analysis of *G. uralensis* candidate genes, significant enrichment of GO terms related to carbohydrate metabolic pathways and biosynthesis (Figure 6), including carbohydrate metabolic process (GO: 0005975, GO: 0009100, GO: 0070085), carbohydrate biosynthetic process (GO:0016051), glucose catabolic process (GO:0006007), carbohydrate derivative biosynthetic process (GO:1901137), monosaccharide biosynthetic process (GO:0046364), hexose catabolic process (GO:0019320), single-organism carbohydrate metabolic process (GO:0044723), hexose biosynthetic process (GO:0019319), was in line with the findings reported by Shimoyama et al. (2020). Multiple studies (Ho et al., 2001; Li et al., 2006) have shown that sucrose (polysaccharides), glucose (monosaccharides), and fructose (monosaccharides) play a central role at the cellular and metabolic levels in plants. These molecules are involved in response to abiotic stress, serve as a nutrient, and function as metabolite signaling molecules.

Significant enrichment of multiple GO terms related to carbohydrate metabolic pathways and biosynthesis could be due to the arid habitat of samples. Accumulation of water-soluble carbohydrates is widely considered to be an adaptive response of plants to drought stress since water-soluble carbohydrates can act as osmotic agents to maintain the enlargement of leaf cells, protect the integrity of cell membranes, and prevent protein denaturation (Bartels and Sunkar, 2005; Verslues et al., 2006). Xue et al. (2008) dissected the coordination of the regulation of key enzyme genes involved in carbon fixation and hexose and fructan accumulation. This elucidated the molecular mechanism of altered carbohydrate metabolism in wheat adapted to drought stress at the transcriptional level. The cellular homeostasis that regulates the cell numbers in a free-living population can activate metabolic functions and biosynthetic pathways that may enhance the *G. uralensis* population’s growth and resistance to drought stress (Bartholomew, 1964; Xue et al., 2008). We hypothesize that the *G. uralensis* population’s metabolic and biosynthesis-related gene families may have expanded rapidly to adapt to the environment and its drought stress. This study also showed that the C levels were significantly higher in *G. uralensis* from the central region (Table 1). This finding was consistent with the significantly enriched GO terms related to biosynthesis and carbohydrate metabolism in the central population. This indicates that the carbon element generated through photosynthesis was actively involved in maintaining the metabolic balance of carbohydrates. Although the regulatory mechanisms involved in carbohydrate metabolism and synthesis need to be further investigated to understand the mechanisms of action, our study provides a reference for the direction of population evolution and environmental adaptation of arid plants.

Bioactive components in medicinal plants are the byproducts of physiological activities during the plant growth phase, which is based on the coevolution of genes and the environment (Li and Wu, 2018). The content of bioactive components in the medicinal plant is the material basis of its clinical efficacy, and these bioactive components are crucial factors for determining the quality of medicinal materials. This study revealed that acyl-CoA dehydrogenase activity (GO: 0003995; Gene name: Glyur000345s00015647) was significantly enriched in eastern (UW) and southern (YW), but not in the central (SW) population of *G. uralensis* (Figure 6). This data could be studied further to discern the genetic differentiation and environmental adaptation of the *G. uralensis* population. Acetyl-coA is a common precursor for the biosynthesis of liquiritin (C_21_H_22_O_9_, flavonoids), glycyrrhizic acid (C_42_H_62_O_16_, triterpenoid saponins) and a series of key genes, such as the chalcone synthase gene (CHS). Previous studies (Shirazi et al., 2019; Wang et al., 2020) have shown that the expression of functional genes was closely related to the accumulation of bioactive components including liquiritin and glycyrrhizic acid in *G. uralensis* plants. The findings of the current study provide a theoretical basis for the protection of wild medicinal plant resources and the breeding of excellent varieties.

Fatty acids (FA), whether as part of a molecule or individually, have a variety of functions in cells, from structural “building blocks” of cell membranes to providers of energy and signaling molecules (Nian and Chen, 2012). The study of FA and its metabolism is important in many research fields, including biology, bacteriology, ecology, human nutrition and health. In this study, KEGG pathway analysis revealed alpha-Linolenic acid metabolism, Biosynthesis of unsaturated fatty acids and Fatty acid degradation were significantly enriched in the eastern and southern of *G. uralensis* population (Table 4), which indicated that functional genes related to biochemical metabolic pathways and signal transduction pathways play an important role in the population differentiation of *G. uralensis*. During long periods of natural selection, plants have developed unique metabolic pathways to cope with different habitats. We hypothesize that these metabolic pathways are closely related to the growth and environmental strategies of licorice, which means that further study of these metabolic pathways may provide important insights into the adaptive evolution of plants.

In conclusion, this study provided new insights on population genetic diversity, genetic structure, and population adaptation of the medicinal plants. There were significant geographical differences in nutrient concentration and photosynthetic of *G. uralensis* leaves at the physiological level. At the genomic level, the genetic structure of *G. uralensis* populations showed significant genetic differentiation with clearly divided into Central, Eastern and Southern. As per the outcomes of this study, many GO terms related to carbohydrate metabolic pathways and biosynthesis play an important role in the population differentiation of *G. uralensis* in central, southern and eastern regions. Further research will be required to characterize the mechanisms by which these GO terms are involved in the population adaptation of plant.

## Data Availability Statement

The data presented in the study are deposited in the National Center for Biotechnology Information (NCBI) repository, accession number PRJNA 730103.

## Author Contributions

XP, HD, and TZ conceived and designed the research project. HD and TZ performed methodology, data analysis, investigated and were major contributor in writing the manuscript, have contributed equally to this work, and share first authorship. YL, GL, and LZ collected leaf samples and modified the manuscript. All authors read and approved the final manuscript.

## Conflict of Interest

The authors declare that the research was conducted in the absence of any commercial or financial relationships that could be construed as a potential conflict of interest.

## Publisher’s Note

All claims expressed in this article are solely those of the authors and do not necessarily represent those of their affiliated organizations, or those of the publisher, the editors and the reviewers. Any product that may be evaluated in this article, or claim that may be made by its manufacturer, is not guaranteed or endorsed by the publisher.

## Footnotes

1
https://doi.org/10.1111/tpj.13385

2
www.openbioinformatics.org/annovar/

3
https://dataview.ncbi.nlm.nih.gov/object/PRJNA730103?reviewer=2ju40n6b4q1f5b7lkahvar4rsh

4
http://treesoft.sourceforge.net/treebest.shtml

5
http://cnsgenomics.com/software/gcta/#Overview

6
http://www.r-project.org/

7
http://www.geneontology.org/
