# Supplementary material for: Population Evolution, Genetic Diversity and Structure of the Medicinal Legume, Glycyrrhiza uralensis and the Effects of Geographical Distribution on Leaves Nutrient Elements and Photosynthesis
Source: Front Plant Sci. 2022 Jan 7;12:708709. doi: 10.3389/fpls.2021.708709 (PMC8782460; doi:10.3389/fpls.2021.708709)
Supplement: Supplementary file 1 [file Data_Sheet_1.docx]

# Supplementary Data

## Supplementary Table

**Supplementary table S1** Effects of geographical location on leaf photosynthesis in *G. uralensis*

|  | **Fv/Fm** | **Y(II)** | **NPQ** | **ETR(I)** | **Y(NO)** | **Y(NPQ)** | **ETR(II)** |
| --- | --- | --- | --- | --- | --- | --- | --- |
| UW | 0.808±0.003 | 0.245±0.028 | 1.645±0.109 | **47.533±4.899 b** | 0.287±0.023 | 0.468±0.007 | **23.367±2.734 b** |
| SW | 0.825±0.006 | 0.342±0.028 | 1.221±0.026 | **38.367±2.293 b** | 0.296±0.010 | 0.362±0.018 | **26.300±2.107 b** |
| YW | 0.819±0.010 | 0.269±0.034 | 1.823±0.278 | **129.800±10.808 a** | 0.262±0.014 | 0.469±0.048 | **82.900±10.473 a** |

Description: UW, SW, YW represents *G. uralensis* of eastern, central and southern regions, respectively. Different letters indicated statistically significant difference (P < 0.05).

**Supplementary table S2** Sequencing data statistics of 60 samples

| **Location** | **Sample** | **Raw Base(bp)** | **Clean Base(bp)** | **Effective Rate(%)** | **Error Rate(%)** | **Q20(%)** | **Q30(%)** | **GC Content(%)** |
| --- | --- | --- | --- | --- | --- | --- | --- | --- |
| **Central region** | SW-1 | 2,887,263,600 | 2,877,639,000 | 99.67 | 0.03 | 95.55 | 89.34 | 37.58 |
|  | SW-2 | 2,649,561,300 | 2,641,192,200 | 99.68 | 0.03 | 96.45 | 91.32 | 37.69 |
|  | SW-3 | 3,250,526,700 | 3,241,147,200 | 99.71 | 0.03 | 95.91 | 90.01 | 37.67 |
|  | SW-4 | 2,621,440,500 | 2,612,808,900 | 99.67 | 0.03 | 96.24 | 90.91 | 37.94 |
|  | SW-5 | 2,364,714,900 | 2,357,756,700 | 99.71 | 0.03 | 95.83 | 89.89 | 37.67 |
|  | SW-6 | 2,459,403,000 | 2,452,605,600 | 99.72 | 0.03 | 95.77 | 89.78 | 37.74 |
|  | SW-7 | 2,696,587,200 | 2,687,345,100 | 99.66 | 0.03 | 95.82 | 89.91 | 37.95 |
|  | SW-8 | 2,894,432,100 | 2,884,773,900 | 99.67 | 0.03 | 95.78 | 89.91 | 37.77 |
|  | SW-9 | 2,267,820,000 | 2,260,441,800 | 99.67 | 0.03 | 95.64 | 89.54 | 38.08 |
|  | SW-10 | 2,977,839,900 | 2,963,919,900 | 99.53 | 0.03 | 95.77 | 89.87 | 37.66 |
|  | SW-11 | 2,505,557,400 | 2,497,407,300 | 99.67 | 0.03 | 95.66 | 89.54 | 37.62 |
|  | SW-12 | 2,401,095,300 | 2,393,552,400 | 99.69 | 0.03 | 95.76 | 89.72 | 37.49 |
|  | SW-13 | 2,266,107,000 | 2,258,078,100 | 99.65 | 0.03 | 95.71 | 89.71 | 38.41 |
|  | SW-14 | 2,964,185,700 | 2,952,234,000 | 99.6 | 0.03 | 95.81 | 89.88 | 37.88 |
|  | SW-15 | 2,294,648,700 | 2,287,864,200 | 99.7 | 0.03 | 96.06 | 90.33 | 39.32 |
|  | SW-16 | 2,533,909,800 | 2,523,389,100 | 99.58 | 0.03 | 96 | 90.12 | 37.08 |
|  | SW-17 | 2,518,152,600 | 2,510,004,600 | 99.68 | 0.03 | 95.99 | 90.18 | 37.77 |
|  | SW-18 | 2,699,998,200 | 2,691,675,900 | 99.69 | 0.03 | 95.71 | 89.62 | 37.59 |
|  | SW-19 | 2,385,682,800 | 2,377,636,500 | 99.66 | 0.04 | 94.39 | 87.12 | 37.46 |
|  | SW-20 | 2,257,808,100 | 2,250,056,400 | 99.66 | 0.03 | 95.78 | 89.83 | 37.81 |
| **Eastern region** | UW- 1 | 2,226,308,400 | 2,220,097,200 | 99.72 | 0.03 | 96.17 | 90.56 | 37.64 |
|  | UW-2 | 2,298,470,700 | 2,288,795,100 | 99.58 | 0.03 | 95.45 | 89.35 | 38.4 |
|  | UW-3 | 2,414,485,500 | 2,404,146,600 | 99.57 | 0.03 | 95.4 | 89.23 | 37.93 |
|  | UW-4 | 2,655,834,600 | 2,646,945,600 | 99.67 | 0.03 | 95.5 | 89.34 | 37.72 |
|  | UW-5 | 2,418,028,200 | 2,411,746,800 | 99.74 | 0.03 | 95.19 | 88.73 | 37.92 |
|  | UW-6 | 2,169,854,700 | 2,160,509,400 | 99.57 | 0.03 | 95.69 | 89.58 | 37.2 |
|  | UW-7 | 2,623,161,300 | 2,614,860,300 | 99.68 | 0.03 | 95.77 | 89.78 | 37.25 |
|  | UW-8 | 2,540,643,600 | 2,530,617,600 | 99.61 | 0.03 | 95.72 | 89.81 | 37.97 |
|  | UW-9 | 2,192,171,400 | 2,183,838,300 | 99.62 | 0.03 | 95.75 | 89.81 | 38.05 |
|  | UW-10 | 2,696,267,700 | 2,686,690,800 | 99.64 | 0.03 | 95.56 | 89.43 | 37.89 |
|  | UW-11 | 2,452,836,000 | 2,444,272,800 | 99.65 | 0.03 | 95.49 | 89.28 | 37.25 |
|  | UW-12 | 2,765,637,300 | 2,756,159,400 | 99.66 | 0.03 | 95.56 | 89.44 | 37.6 |
|  | UW-13 | 2,498,313,900 | 2,489,253,600 | 99.64 | 0.03 | 95.56 | 89.45 | 37.41 |
|  | UW-14 | 2,097,154,200 | 2,090,287,200 | 99.67 | 0.03 | 95.66 | 89.61 | 37.84 |
|  | UW-15 | 2,586,031,800 | 2,578,629,900 | 99.71 | 0.03 | 95.73 | 89.73 | 37.3 |
|  | UW-16 | 2,389,867,800 | 2,377,618,800 | 99.49 | 0.04 | 94.78 | 88 | 37.92 |
|  | UW-17 | 2,016,006,300 | 2,009,647,800 | 99.68 | 0.03 | 95.77 | 89.78 | 37.41 |
|  | UW-18 | 2,525,801,400 | 2,514,237,300 | 99.54 | 0.03 | 95.34 | 89.06 | 37.95 |
|  | UW-19 | 3,011,900,100 | 3,001,004,100 | 99.64 | 0.03 | 95.9 | 90.28 | 38.3 |
|  | UW-20 | 2,482,888,500 | 2,471,195,700 | 99.53 | 0.03 | 95.3 | 89.06 | 38.15 |
| **Southern region** | YW-1 | 2,347,063,200 | 2,339,755,500 | 99.69 | 0.03 | 95.69 | 89.7 | 37.34 |
|  | YW-2 | 2,108,347,200 | 2,102,762,100 | 99.74 | 0.03 | 95.72 | 89.64 | 37.17 |
|  | YW-3 | 2,480,458,800 | 2,471,571,000 | 99.64 | 0.03 | 95.62 | 89.49 | 37.38 |
|  | YW-4 | 2,660,202,000 | 2,647,042,800 | 99.51 | 0.03 | 96.09 | 90.16 | 36.38 |
|  | YW-5 | 2,442,576,900 | 2,435,360,100 | 99.7 | 0.03 | 95.55 | 89.33 | 37.26 |
|  | YW-6 | 2,983,531,500 | 2,974,847,400 | 99.71 | 0.03 | 95.95 | 90.05 | 37.06 |
|  | YW-7 | 3,326,453,700 | 3,317,166,300 | 99.72 | 0.03 | 96.52 | 91.3 | 36.89 |
|  | YW-8 | 2,093,382,000 | 2,087,397,900 | 99.71 | 0.03 | 95.95 | 90.05 | 36.9 |
|  | YW-9 | 2,452,304,400 | 2,442,898,200 | 99.62 | 0.03 | 96.57 | 91.58 | 37.67 |
|  | YW-10 | 2,331,932,700 | 2,323,452,600 | 99.64 | 0.03 | 95.94 | 90.08 | 37.3 |
|  | YW-11 | 2,555,917,500 | 2,546,625,600 | 99.64 | 0.03 | 95.81 | 89.86 | 37.37 |
|  | YW-12 | 2,576,334,600 | 2,566,447,800 | 99.62 | 0.03 | 95.78 | 89.8 | 37.61 |
|  | YW-13 | 2,633,391,300 | 2,624,073,900 | 99.65 | 0.03 | 95.7 | 89.64 | 37.38 |
|  | YW-14 | 2,503,957,200 | 2,493,743,700 | 99.59 | 0.03 | 95.74 | 89.73 | 37.41 |
|  | YW-15 | 2,385,383,400 | 2,376,771,900 | 99.64 | 0.03 | 95.9 | 89.91 | 37.03 |
|  | YW-16 | 2,058,218,100 | 2,051,675,400 | 99.68 | 0.03 | 95.88 | 89.95 | 37.11 |
|  | YW-17 | 2,056,098,900 | 2,046,348,300 | 99.53 | 0.03 | 95.72 | 89.61 | 37.52 |
|  | YW-18 | 2,651,975,400 | 2,640,702,900 | 99.57 | 0.03 | 95.57 | 89.41 | 37.52 |
|  | YW-19 | 2,715,485,100 | 2,706,085,500 | 99.65 | 0.03 | 95.94 | 90.1 | 37.3 |
|  | YW-20 | 2,277,338,700 | 2,270,127,300 | 99.68 | 0.03 | 95.95 | 90.03 | 37.05 |

Description: Sample name: UW, SW, YW refers to *G. uralensis* of eastern, central and southern regions, respectively, number represents the replicate number. Raw Base refers to raw data production (bp); Clean Base refers to the amount of effective data after filtering (bp); Effective Rate refers to the ratio of clean data to raw data (%); Error Rate refers to base error rate (%); Q20 and Q30 respectively refers to the percentage of bases whose Phred value is greater than 20 and 30 in the total base (%); GC Content refers to the sum of the number of bases G and C as a percentage of the total number of bases (%).

**Supplementary table S3** Sequencing depth and coverage of each sample

| **Location** | **Sample** | **Total reads** | **Mapped reads** | **Mapping rate(%)** | **Average depth(**X**)** | **Coverage at least 1**X **(%)** | **Coverage at least 4**X **(%)** |
| --- | --- | --- | --- | --- | --- | --- | --- |
| **Central region** | SW-1 | 16350704 | 14931989 | 91.32 | 8.98 | 87.5 | 56.48 |
|  | SW-2 | 17607948 | 15924944 | 90.44 | 9.33 | 87.97 | 58.86 |
|  | SW-3 | 21607648 | 19616678 | 90.79 | 10.3 | 89.45 | 68.32 |
|  | SW-4 | 17418726 | 15879552 | 91.16 | 9.22 | 87.97 | 58.79 |
|  | SW-5 | 15718378 | 14253446 | 90.68 | 8.85 | 87.05 | 54.13 |
|  | SW-6 | 16350704 | 14931989 | 91.32 | 8.98 | 87.5 | 56.48 |
|  | SW-7 | 17915634 | 15838223 | 88.4 | 9.26 | 87.89 | 58.86 |
|  | SW-8 | 19231826 | 17187518 | 89.37 | 9.4 | 88.38 | 64.64 |
|  | SW-9 | 15069612 | 13280277 | 88.13 | 8.78 | 86.01 | 50.01 |
|  | SW-10 | 19759466 | 17912577 | 90.65 | 9.79 | 88.96 | 64.74 |
|  | SW-11 | 16649382 | 15009096 | 90.15 | 8.99 | 87.88 | 57.39 |
|  | SW-12 | 15069612 | 13280277 | 88.13 | 8.78 | 86.01 | 50.01 |
|  | SW-13 | 15053854 | 13207800 | 87.74 | 8.73 | 85.81 | 49.61 |
|  | SW-14 | 19681560 | 17641882 | 89.64 | 9.76 | 88.81 | 63.93 |
|  | SW-15 | 15252428 | 12297266 | 80.62 | 8.43 | 85.58 | 47.52 |
|  | SW-16 | 16822594 | 14851146 | 88.28 | 9.43 | 86.94 | 53.68 |
|  | SW-17 | 16733364 | 14964935 | 89.43 | 9.22 | 87.03 | 55.45 |
|  | SW-18 | 17944506 | 15798139 | 88.04 | 9.37 | 87.93 | 58.8 |
|  | SW-19 | 15850910 | 14341449 | 90.48 | 8.78 | 87.63 | 55.61 |
|  | SW-20 | 15000376 | 13473104 | 89.82 | 8.61 | 86.74 | 52.39 |
| **Eastern region** | UW-1 | 14800648 | 13418919 | 90.66 | 8.72 | 85.48 | 49.54 |
|  | UW-2 | 15258634 | 13919725 | 91.23 | 8.63 | 86.06 | 51.56 |
|  | UW-3 | 16027644 | 14579271 | 90.96 | 8.76 | 87.85 | 54.53 |
|  | UW-4 | 17646304 | 16056492 | 90.99 | 9.12 | 87.99 | 59.94 |
|  | UW-5 | 16078312 | 14700519 | 91.43 | 8.75 | 86.98 | 55.25 |
|  | UW-6 | 14403396 | 13038958 | 90.53 | 8.51 | 85.9 | 50.92 |
|  | UW-7 | 17432402 | 15740606 | 90.3 | 9.2 | 87.54 | 58.84 |
|  | UW-8 | 16870784 | 15265411 | 90.48 | 8.98 | 87.26 | 56.65 |
|  | UW-9 | 14558922 | 13306049 | 91.39 | 8.53 | 86 | 50.24 |
|  | UW-10 | 17911272 | 16400237 | 91.56 | 9.15 | 88.32 | 61.56 |
|  | UW-11 | 16295152 | 14943647 | 91.71 | 8.8 | 87.5 | 58.57 |
|  | UW-12 | 18374396 | 16740899 | 91.11 | 9.34 | 88.31 | 62.4 |
|  | UW-13 | 16595024 | 15125473 | 91.14 | 8.92 | 87.39 | 57.78 |
|  | UW-14 | 13935248 | 12745343 | 91.46 | 8.32 | 85.81 | 49.98 |
|  | UW-15 | 17190866 | 15448696 | 89.87 | 9.15 | 87.33 | 57.62 |
|  | UW-16 | 15850792 | 14266687 | 90.01 | 8.75 | 86.81 | 53.34 |
|  | UW-17 | 13397652 | 12286499 | 91.71 | 8.27 | 85.32 | 47.24 |
|  | UW-18 | 16761582 | 15319296 | 91.4 | 8.98 | 87 | 57.42 |
|  | UW-19 | 20006694 | 17791401 | 88.93 | 9.49 | 88.55 | 64.37 |
|  | UW-20 | 16474638 | 14973897 | 90.89 | 8.9 | 86.9 | 55.47 |
| **Southern region** | YW-1 | 19832316 | 17766215 | 89.58 | 9.86 | 88.53 | 65.21 |
|  | YW-2 | 14018414 | 12697927 | 90.58 | 8.49 | 85.73 | 49.06 |
|  | YW-3 | 16477140 | 14753057 | 89.54 | 8.96 | 87.07 | 56.22 |
|  | YW-4 | 17646952 | 16223155 | 91.93 | 9.69 | 87.78 | 60.52 |
|  | YW-5 | 16235734 | 14549716 | 89.62 | 9 | 87.15 | 55.03 |
|  | YW-6 | 19832316 | 17766215 | 89.58 | 9.86 | 88.53 | 65.21 |
|  | YW-7 | 22114442 | 19569373 | 88.49 | 10.5 | 89.06 | 68.9 |
|  | YW-8 | 13915986 | 12381282 | 88.97 | 8.7 | 85.15 | 45.96 |
|  | YW-9 | 16285988 | 14687419 | 90.18 | 9.06 | 86.59 | 55.44 |
|  | YW-10 | 15489684 | 13683599 | 88.34 | 9 | 85.73 | 50.66 |
|  | YW-11 | 16977504 | 15137189 | 89.16 | 9.17 | 87.19 | 56.73 |
|  | YW-12 | 13642322 | 11828561 | 86.7 | 8.8 | 83.73 | 42.28 |
|  | YW-13 | 17493826 | 15456985 | 88.36 | 9.31 | 87.1 | 57.62 |
|  | YW-14 | 16624958 | 14785717 | 88.94 | 9.17 | 86.54 | 55.15 |
|  | YW-15 | 15845146 | 13900094 | 87.72 | 9.15 | 85.83 | 51.26 |
|  | YW-16 | 13677836 | 12422609 | 90.82 | 8.38 | 85.7 | 49.07 |
|  | YW-17 | 13642322 | 11828561 | 86.7 | 8.8 | 83.73 | 42.28 |
|  | YW-18 | 17604686 | 15629450 | 88.78 | 9.28 | 87.47 | 58.42 |
|  | YW-19 | 18040570 | 16106802 | 89.28 | 9.5 | 87.37 | 59.34 |
|  | YW-20 | 15134182 | 13373448 | 88.37 | 8.92 | 85.71 | 50.37 |

Description: Sample name: UW, SW, YW refers to *G. uralensis* of eastern, central and southern regions, respectively, number represents the replicate number. Total reads refers to the total number of reads from effective sequencing data; Mapped reads refers to the number of reads Mapped into reference genome (including single - ended comparison and double - ended comparison); Mapping rate refers to the number of reads compared into the reference genome divided by the number of reads with effective sequencing data (%); Average depth refers to the total number of bases compared to the reference genome divided by the genome size (X); Coverage at least 1 X refers to the percentage of the genome with at least one base covering the reference genome (%); Coverage at least 4 X refers to the percentage of the genome with at least four base covering the reference genome (%).

**Supplementary table S4** *G. uralensis* Population genetic diversity analysis

| **Population** | **Ho** | **He** | **π** |
| --- | --- | --- | --- |
| UW | 0.443 | 0.303 | 0.307 |
| SW | 0.492 | 0.324 | 0.329 |
| YW | 0.436 | 0.300 | 0.304 |

Description: Population: UW, SW, YW refers to *G. uralensis* population of eastern, central and southern regions, respectively. The higher the observed heterozygosity (Ho), the expected heterozygosity (He) and Nucleotide diversity analysis (π), the higher the heterozygosity, indicating the higher the genetic diversity within the population. Values range from 0 (indicating no polymorphism) to 1 (indicating an infinite number of alleles with the same frequency, which is a limit).

## Supplementary Figures


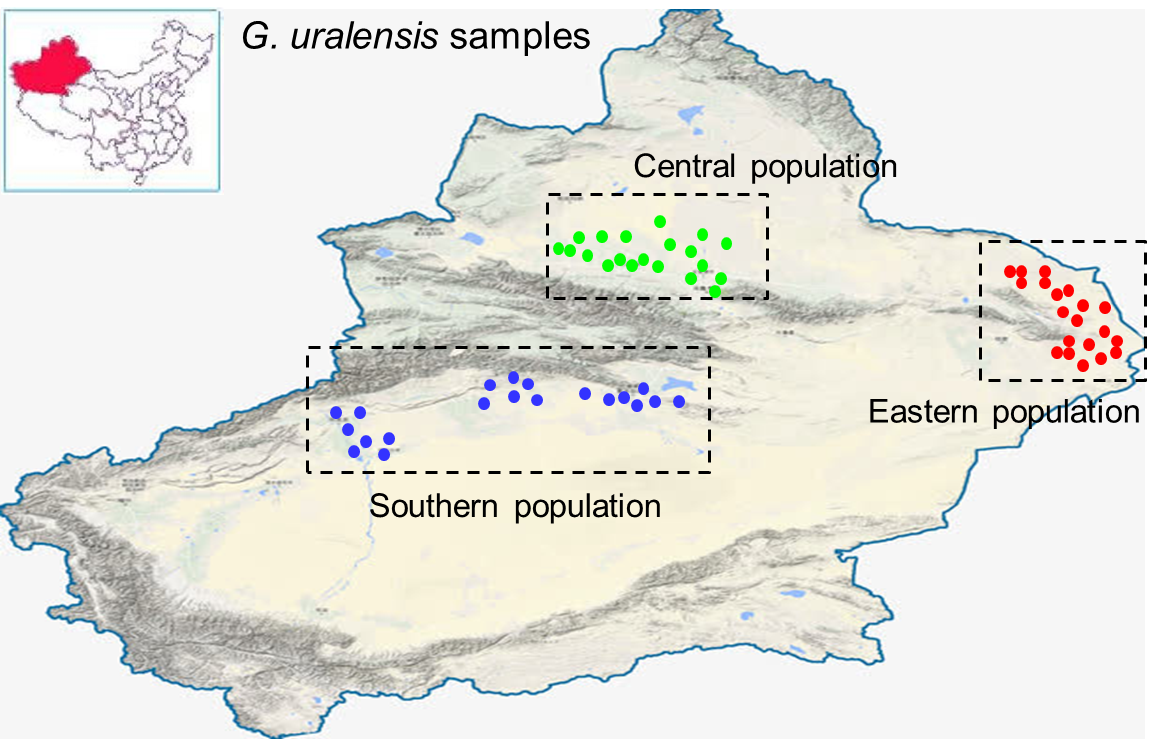


**Supplementary Figure 1.** Geographical distribution of *G. uralensis*


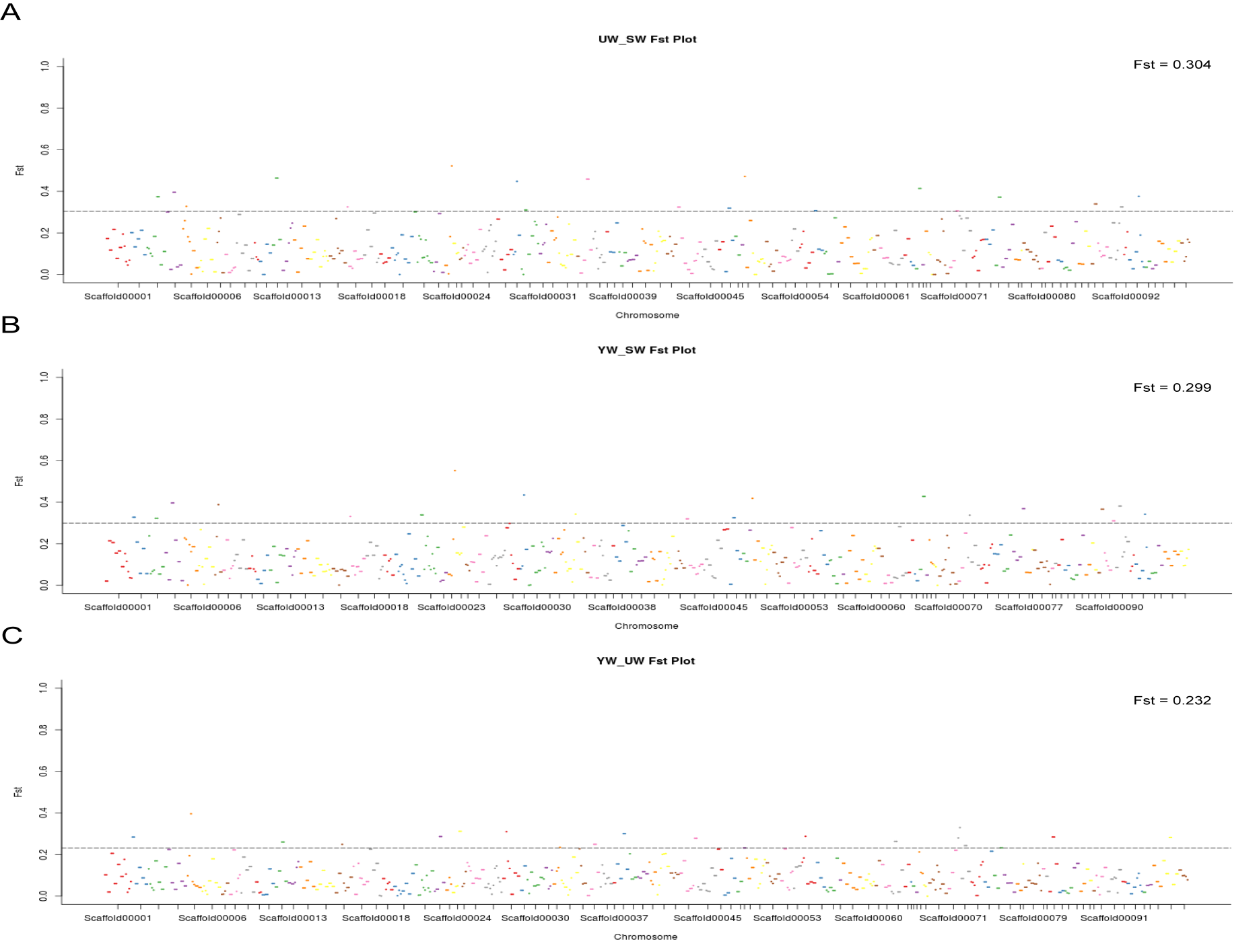


**Supplementary Figure 2.** The distribution of Fst in the genome of *G. uralensis* populations

Description: The abscissa represents the different chromosome names, the ordinate represents the Fst value in the corresponding chromosome window. The other two dotted lines represent the two selection thresholds (top 5%). (a), (b), (c) represents *G. uralensis* of central, eastern and southern regions, respectively.
